# Integrative Meta-Analysis Identifies Epithelial–Mesenchymal Transition Gene Signatures as Key Determinants of Ovarian Cancer Progression and Treatment Outcome

**DOI:** 10.3390/ijms27052149

**Published:** 2026-02-25

**Authors:** Matteo Cassandri, Paola Pontecorvi, Fabrizio Cece, Simona Camero, Giada Mele, Enrico Romano, Simona Ceccarelli, Roberto Rizzi, Francesco Marampon, Antonio Angeloni, Cinzia Marchese, Francesca Megiorni

**Affiliations:** 1 Department of Experimental Medicine, Sapienza University of Rome, 00161 Rome, Italy; matteo.cassandri@uniroma1.it (M.C.); fabrizio.cece@uniroma1.it (F.C.); simona.ceccarelli@uniroma1.it (S.C.); cinzia.marchese@uniroma1.it (C.M.); 2 Department of Medical-Surgical Sciences and Biotechnologies, Sapienza University of Rome, Viale dell’Università 30, 00185 Rome, Italy; paola.pontecorvi@uniroma1.it; 3 Department of Life Sciences, Health and Health Professions, Link Campus University, 00165 Rome, Italy; s.camero@unilink.it; 4 Department of Radiological, Oncological and Pathological Sciences, Sapienza University of Rome, 00161 Rome, Italy; giada.mele@uniroma1.it; 5 Department of Sense Organs, Sapienza University of Rome, 00161 Rome, Italy; enrico.romano@uniroma1.it; 6 Department of Well-being, Health and Environmental Sustainability, Sapienza University of Rome, 02100 Rieti, Italy; roberto.rizzi@uniroma1.it (R.R.); francesco.marampon@uniroma1.it (F.M.); antonio.angeloni@uniroma1.it (A.A.)

**Keywords:** Ovarian cancer, epithelial–mesenchymal transition, meta-analysis, The Cancer Genome Atlas

## Abstract

Ovarian cancer (OC) remains one of the most lethal gynecologic malignancies, with nearly 80% of patients diagnosed at advanced stages due to the absence of early symptoms and the nonspecific nature of later clinical manifestations. This highlights the urgent need for robust molecular biomarkers that can refine patient stratification and guide personalized therapeutic approaches. A major determinant of OC aggressiveness is the epithelial-to-mesenchymal transition (EMT), a transcriptionally driven program that represses epithelial identity while promoting mesenchymal traits, thereby enhancing invasion, dissemination, recurrence, and resistance to therapy. EMT dysregulation is widespread in OC and fuels tumor heterogeneity, metastatic spread, and chemoresistance. To investigate the contribution of EMT-related genes in OC biology, we analyzed whole-genome sequencing and RNA-seq data from 419 patients in The Cancer Genome Atlas (TCGA) Pan-Cancer Atlas, assessing their genomic and transcriptomic alterations. We integrated these findings with transcriptomic and drug-sensitivity data from the CTRPv2 portal, performing Pearson correlation analyses to identify therapeutic vulnerabilities associated with EMT gene expression. Our analysis identifies recurrent genomic and transcriptomic alterations across several EMT-associated genes. Notably, we identified a four-EMT gene signature (EFNA1, OVOL2, GATA3, and DSG2) whose expression correlates with differential sensitivity to VEGFR and EGFR inhibitors in OC cell lines. Overall, these results suggest that EMT-driven molecular changes contribute to the onset and progression of OC and highlight a subset of EMT genes as promising predictive biomarkers for targeted therapy responses.

## 1. Introduction

Ovarian cancer (OC) is among the leading causes of malignancy-associated death in women worldwide. In Italy, the 2023 report by the Italian Association of Cancer Registries and Medical Oncology stated that OC affects around 6000 women annually, with a five-year survival rate of 43%. Survival varies by ethnicity, genetic predisposition, and geographic location. Most cases are diagnosed in women aged 50–70 years, with approximately 80% of patients presenting at an advanced stage (FIGO stage III or IV), mainly due to its asymptomatic early stages and nonspecific symptoms in later phases. A family history of ovarian or breast cancer and specific genetic mutations further increase the risk of developing this type of tumor. OC is a heterogeneous disease comprising distinct histological subtypes, including epithelial carcinomas (more than 90%), sex cord-stromal tumors (~5%), and germ cell tumors (1–2%). Among epithelial tumors, high-grade serous ovarian carcinoma (HGSOC) is the most prevalent subtype and is characterized by marked chromosomal instability, extensive copy number variations (CNVs), and frequent TP53, BRCA1, and BRCA2 mutations [1]. Emerging evidence highlights the oncogenic role of the MYC family in OC [2]. Amplification or overexpression of MYC has been reported in a significant proportion of ovarian tumors, where it contributes to uncontrolled proliferation, altered metabolism, and transcriptional reprogramming. Analyses from The Cancer Genome Atlas (TCGA) reported MYC amplification in approximately 30.7% of ovarian tumors [3], while a study of 276 HGSOC samples found MYC amplification in 19.2% of cases [4]. Primary treatments for OC include debulking surgery and systemic chemotherapy, often combined with targeted therapies, such as the anti-VEGF agent, bevacizumab or PARP inhibitors, used either as part of first-line therapy or as maintenance when clinically appropriate [5,6,7]. However, cancer relapse is still a major limitation and decreases patient survival and quality of life. Patients frequently exhibit poor prognosis and low five-year survival rates (approximately 30%), mainly due to metastasis, chemoresistance, and rapid tumor recurrence [8,9].

Therefore, the identification of molecular biomarkers that can guide the selection of patients for personalized therapies is urgently needed. In this regard, one should consider that the antitumor effect of chemotherapeutic or targeted treatments largely depends on their ability to interfere with essential cellular processes sustaining tumor growth, including those regulating the epithelial-to-mesenchymal transition (EMT). Throughout tumor progression, OC cells experience various endogenous and exogenous stressors, such as oxidative stress, inflammation, and microenvironmental cues, that promote phenotypic plasticity and EMT activation. EMT is responsible for tumor relapse in several cancers [10]. This process is orchestrated by a complex network of transcription factors, which suppress epithelial markers (e.g., E-cadherin) and induce mesenchymal genes (e.g., N-cadherin and vimentin), facilitating invasion, dissemination, and drug resistance. Importantly, alterations in signaling pathways and transcriptional regulators driving EMT are frequently observed in OC and contribute to tumor heterogeneity, metastatic potential, and chemoresistance [11]. These EMT-associated alterations, combined with genetic and epigenetic instability, promote oncogene activation and/or loss of tumor suppressors, resulting in an aggressive disease phenotype. Nevertheless, little evidence is reported to uncover the EMT-related gene signatures that may serve as valuable prognostic and predictive biomarkers as well as potential therapeutic targets in OC.

Based on this evidence, in the present study, we analyzed the alteration status and expression of EMT-associated genes in a cohort of OC patients retrieved from the TCGA database. Using bioinformatic approaches, we aim to identify dysregulated genes and uncover correlations between the expression of (i) specific EMT-related genes and BRCA1 mutational status; (ii) EMT gene expression patterns and MYC gene alterations; and (iii) an EMT-based signature role in predicting OC responsiveness to VEGFR inhibitors, therapeutic agents currently used in the clinical management of OC patients. The results can be used to develop novel molecular markers for prognosis prediction in patients with OC.

## 2. Results

### 2.1. EMT Gene Mutations in OC Patients

**Figure 1:**
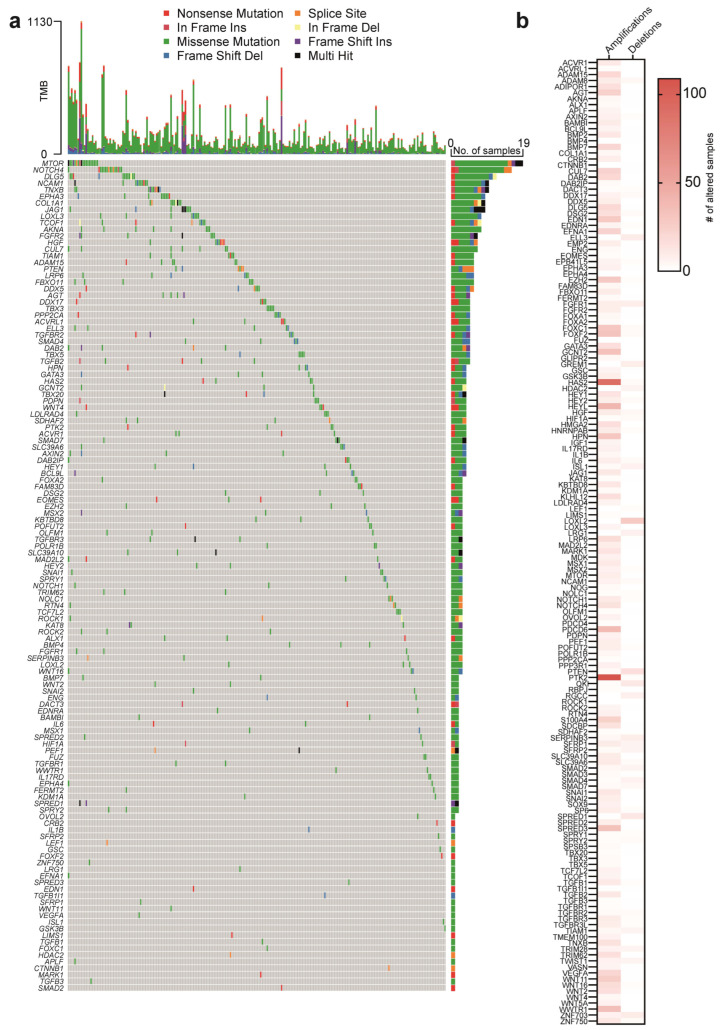
Genetic alterations of EMT genes in OC primary biopsies. (**a**) Onco-plots depicting the genomic alterations of EMT-associated genes in OC patients from TCGA. Tumor Mutational Burden (TMB) values reflect the frequency and types of gene mutations across affected individuals, with specific classes of mutations color-coded according to the legend. Genes are arranged from top to bottom by the decreasing number of alterations present in OC samples, as reported in the right panel. (**b**) Heatmap depicting copy number variations (CNVs; amplifications and deletions) of EMT-associated genes in OC patients.

To assess the impact of EMT-associated genes in OC onset and progression, we selected 398 OC patient’s samples from The Cancer Genome Atlas (TCGA) ovarian serous cystadenocarcinoma dataset, which is almost exclusively composed of HGSOC samples, (Supplementary Table S1) and we focused on 160 coding genes belonging to the human gene set GOBP Epithelial to Mesenchymal Transition (accession code: GO:0001837) (Supplementary Table S2). Firstly, to investigate whether EMT-associated genes exhibit genomic alterations in OC primary tumors, we examined mutation and copy-number profiles. Overall, 126 genes out of 160 analyzed showed alterations in at least one patient. The most common alterations are missense mutations, which have been found in 112 genes out of 160 (Figure 1a). Particularly, 20 genes showed a genomic alteration percentage greater than 1%. Among the most frequently mutated genes were MTOR, NOTCH4, and DLG5, with several OC patients exhibiting particularly high mutation burdens. Interestingly, these genes are characterized not only by missense mutations but also by splice site, frameshift, nonsense, and multi-hit mutations. Missense and truncating mutations represent the majority of coding changes, whereas splice-site or in-frame alterations are comparatively less frequent.

To further explore EMT genes’ genomic alterations in OC patients, we also analyzed CNVs of these genes. Interestingly, almost all the genes in the collection under analysis show amplification in at least one patient (153 genes out of 160); 47 genes were found to be amplified in at least 10 patients (thus with a frequency of at least 2.5%), and 22 genes show an amplification frequency greater than 5% (amplified in at least 20 patients) (Figure 1b). Moreover, 3 genes show an amplification frequency greater than 10%. Specifically, HEYL, HAS2, and PTK2 were found to be amplified in 40, 91, and 109 patients, respectively. On the other hand, deletions were found to be less frequent than amplifications. In fact, we identified 96 genes that show a deletion in at least one patient, and only 4 genes, QKI, SPRED1, PTEN, and LOXL2, were found to have a deletion in at least 10 patients (a frequency of 2.5%). Only LOXL2 showed a deletion frequency greater than 7%, with deletions observed in 28 out of 398 patients. In particular, MTOR, NOTCH4, and DLG5 resulted in being the most mutated genes; HEYL, HAS2, and PTK2 the most amplified genes; while LOXL2 is the only gene that showed a significant deletion frequency. Together, these findings suggest that genomic deregulation of EMT genes is a frequent event in OC patients and may contribute to tumor aggressiveness.

### 2.2. Correlation of EMT Gene Expression in OC Patients

**Figure 2:**
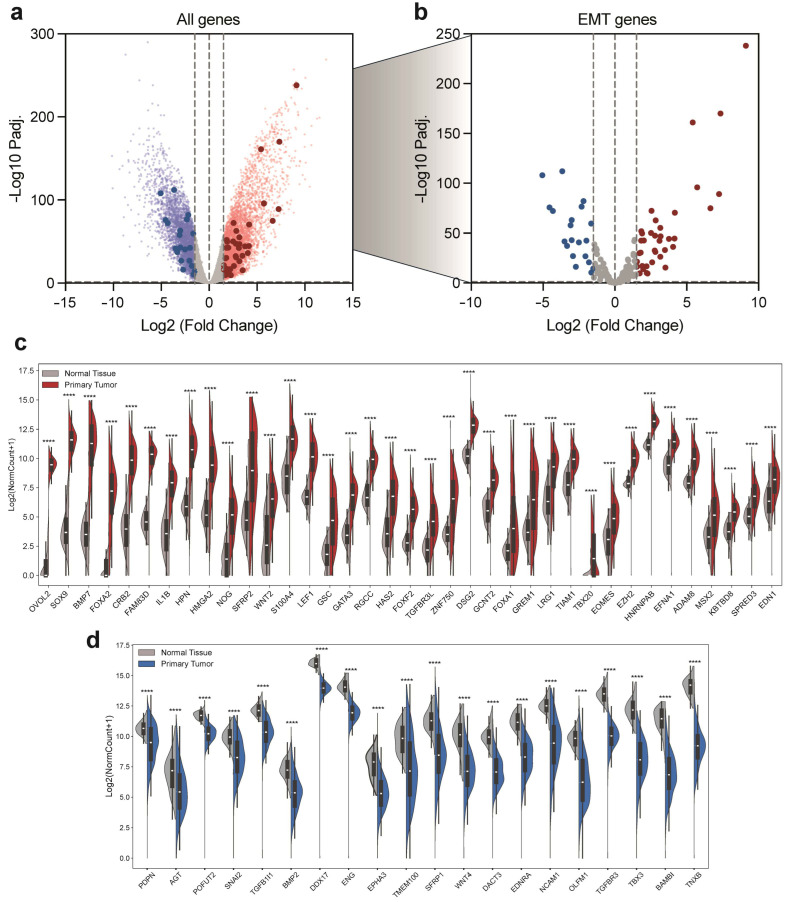
EMT-associated genes differential expression in OC patients compared to normal ovarian tissue. (**a**) Volcano plot depicting differential expression analysis between 419 OC patients from the TCGA database and 88 normal bulk whole-ovary tissue from the GTEx database. (**b**) Volcano plot depicting EMT-associated genes differential expression between 419 OC patients from the TCGA database and 88 normal bulk whole-ovary tissue from the GTEx database. Blue dots represent downregulated genes, while red dots represent upregulated genes. Vertical dash lines represent Log2FC threshold (Log2FC > 1.5 for upregulated genes and Log2FC < 1.5 for downregulated genes). (**c**) Violin plot depicting expression of significantly upregulated EMT genes between OC patients and normal bulk whole-ovary tissue. (**d**) Violin plot depicting expression of significantly downregulated EMT genes between OC patients and normal bulk whole-ovary tissue. *p*-values are reported in the figures. One-way ANOVA multiple comparisons. **** *p* < 0.0001.

To assess whether the observed genomic alterations had a corresponding effect at the expression level, we performed a differential expression analysis by comparing RNA-seq data from 419 OC patients with 88 normal ovarian tissue samples obtained from the Genotype-Tissue Expression (GTEx) database, derived from bulk whole-ovary tissue samples. The analysis revealed that, among the roughly 23,000 genes examined, around 5000 genes were significantly upregulated (Log2FC > 1.5, *p* ≤ 0.05), while approximately 4000 genes were found to be downregulated (Log2FC < −1.5, *p* ≤ 0.05) (Figure 2a). As shown in Figure 2b, EMT genes displayed a widespread and significant dysregulation in tumor samples. Indeed, among these genes, we found 37 EMT-related genes significantly upregulated and 20 significantly downregulated. This subset of dysregulated EMT genes includes both transcriptional regulators (such as SNAI2, SOX9, FOXA1, FOXA2, GATA3, TBX3, TBX20, EOMES, HMGA2, MSX2, ZNF750, and GSC) and signaling modulator (such as BMP2, BMP7, WNT2, WNT4, SFRP1, SFRP2, EDN1, EDNRA, GREM1, NOG, BAMBI, TGFBR3, and TGFB1I1) (Figure 2c,d). Particularly, OVOL2, SOX9, BMP7, FOXA2, CRB2, and FAM83D were the most upregulated genes (Log2FC > 5) (Figure 2b), while the most downregulated genes were TBX3, BAMBI, and TNBX (Log2FC < −4) (Figure 2c). Interestingly, the expression of genes characterized by amplifications or deletions identified in the CNV analysis does not appear to be significantly affected by these genomic alterations. Indeed, among the amplified genes, only HAS2 (Figure 2c) is markedly and significantly upregulated, whereas PTK2, which showed the highest amplification, is only slightly upregulated (Log2FC = 0.65), and HEYL is even mildly downregulated (Log2FC = −1.18). Conversely, among the genes affected by deletions, none is markedly downregulated; only QKI and PTEN show a mild decrease in expression (Log2FC = −0.57 and −0.42, respectively), whilst SPRED1 and LOXL2 show no significant changes in gene expression (*p* > 0.05). Taken together, these findings indicate that the altered expression of EMT genes in OC patients could not be driven by underlying genomic alterations.

### 2.3. Correlation of EMT Gene Expression and BRCA1/BRCA2 Genetic Status and MYC Alterations in OC Patients

**Figure 3:**
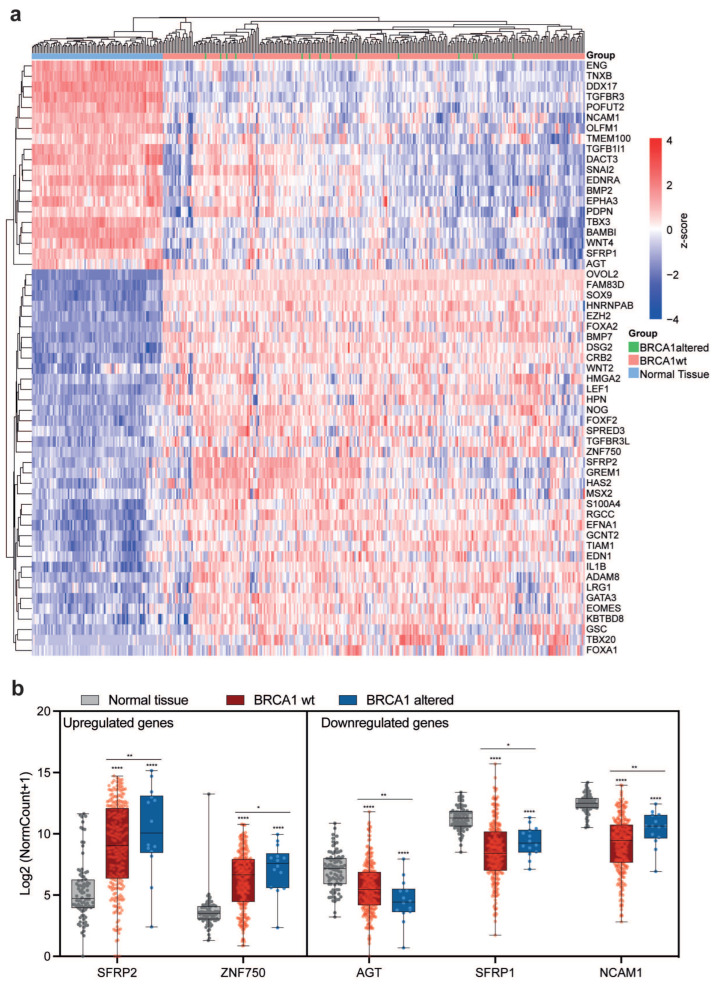
Correlation of EMT gene expression and BRCA1/BRCA2 genetic status. (**a**) Cluster heatmap depicting upregulated and downregulated EMT genes in normal bulk whole-ovary tissue, BRCA1-positive and BRCA1-altered patients. (**b**) Boxplots of significantly and differentially expressed EMT genes between BRCA1-positive and BRCA1-altered patients. *p*-values are reported in the figure. One-way ANOVA multiple comparisons. * *p* < 0.05; ** *p* < 0.01; **** *p* < 0.0001.

Given that genomic alterations in MYC and BRCA1 are frequent in OC patients [12,13], approximately 30% and 6%, respectively, and considering that their role in regulating EMT-related genes is well established in ovarian and other tumor types [14,15], we performed a cluster analysis to assess whether the expression of EMT genes could stratify patients based on MYC and BRCA1 alterations (Figure 3a). Interestingly, the analysis revealed that the expression of EMT genes does not vary significantly between patients with genomic alterations in BRCA1 (Figure 3a). However, some genes do show differential expression between patients with wild-type and altered BRCA1. Among the upregulated genes, SFRP2 and ZNF750 display higher expression levels in patients with BRCA1 alterations. Conversely, among the downregulated genes, while AGT is even more downregulated in BRCA1-altered patients, SFRP1 and NCAM1 exhibit a weaker downregulation compared with normal tissue in BRCA1-altered samples relative to their wild-type counterparts (Figure 3b). This suggests that BRCA-dependent DNA repair deficiency may influence EMT regulation, directly or indirectly, potentially contributing to increased tumor plasticity in OC patients.

**Figure 4:**
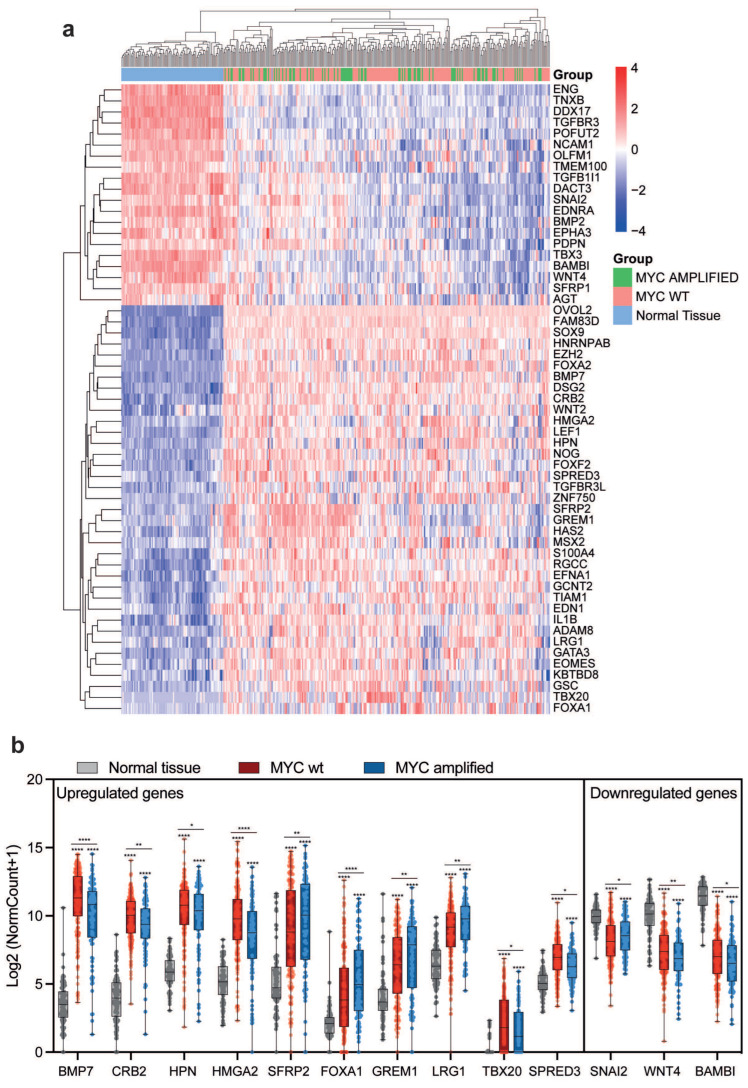
Correlation of EMT Gene Expression and MYC amplification status. (**a**) Cluster heatmap depicting upregulated and downregulated EMT genes in normal bulk whole-ovary tissue, MYC wt, and MYC-amplified patients. (**b**) Boxplots of significantly and differentially expressed EMT genes between MYC wt and MYC-amplified patients. *p*-values are reported in the figure. One-way ANOVA multiple comparisons. * *p* < 0.05; ** *p* < 0.01; **** *p* < 0.0001. Together, these findings indicate that EMT activation in OC is influenced by underlying genetic context, particularly BRCA1/2 loss and MYC amplification.

We also evaluated EMT gene expression according to MYC alteration status. The analysis revealed that, also in this case, the expression of EMT genes is not able to markedly and significantly stratify OC patients based on MYC alterations (Figure 4a). Nevertheless, we observed that among the upregulated genes, BMP7, CRB2, HPN, HMGA2, TBX20, and SPRED3 show lower expression levels in MYC-amplified samples. Conversely, SFRP2, FOXA1, GREM1, and LRG1 display higher upregulation in MYC-amplified samples (Figure 4b). On the other hand, among the downregulated genes, we observed that while SNAI2 shows higher expression levels in patients with MYC amplifications, WNT4 and BAMBI are less downregulated relative to normal tissue in MYC-amplified samples (Figure 4b). Given the known ability of MYC to perturb transcriptional networks and metabolic states, these observations are consistent with MYC as a major driver of EMT transcriptional reprogramming in OC.

### 2.4. EMT Gene Expression and OC Patients’ Overall Survival

**Figure 5:**
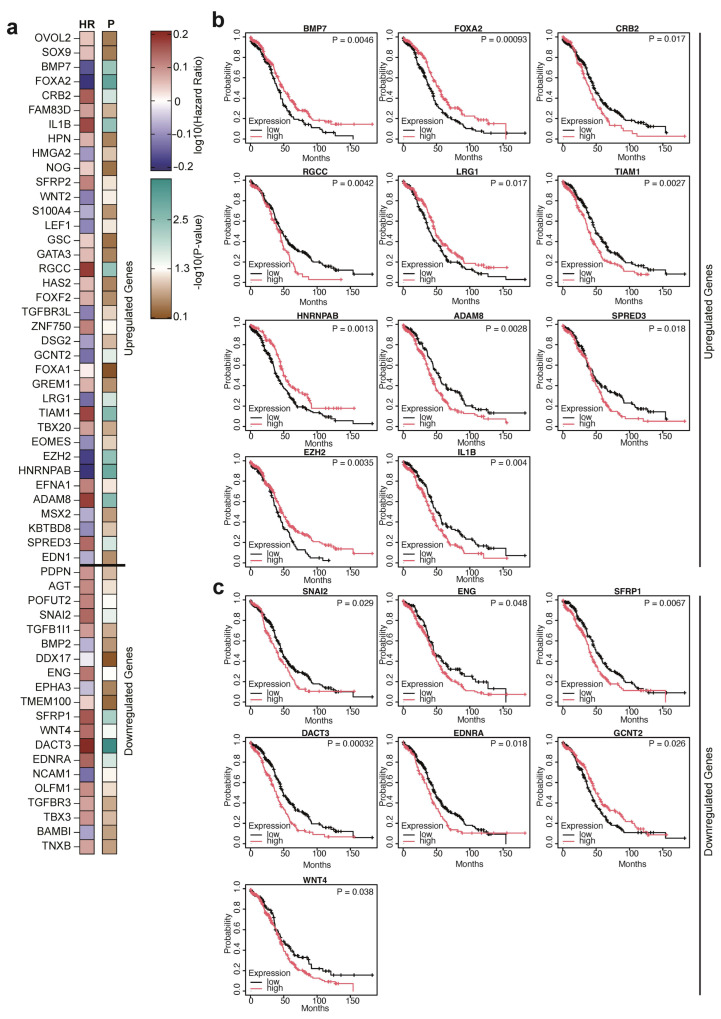
Correlation between EMT-associated genes and OC patients’ overall survival. (**a**) Heatmaps depicting hazard ratio (HR, left) and relative *p*-value (P, right) of EMT genes in OC patients. (**b**) Kaplan-Maier curves depicting overall survival of OC patients stratified on upregulated genes expression. (**c**) Kaplan-Maier curves depicting the overall survival of OC patients stratified by downregulated gene expression. Significant Log-rank *p*-values are reported in the figure.

To determine the prognostic relevance of EMT-associated gene dysregulation, we analyzed the correlation between gene expression and OC patients’ overall survival. As shown in Figure 5a, Hazard Ratios were calculated for the 57 differentially expressed (37 upregulated and 20 downregulated) EMT genes, quantifying the relative risk of death associated with altered expression compared with a reference group.

Interestingly, among the EMT-related genes examined, only 18 genes showed a significant correlation with overall survival. Specifically, Kaplan–Meier curves revealed distinct survival patterns linked to their expression profiles. In particular, we identified a subset of upregulated genes whose high expression correlates with worse prognosis, including CRB2, RGCC, TIAM1, ADAM8, SPRED3, and IL1B. Conversely, FOXA2, BMP7, LRG1, HNRNPAB, and EZH2 are overexpressed in OC patients, but their high expression is associated with better prognosis (Figure 5b). Regarding the downregulated genes, most of them show low expression levels that correlate with improved prognosis, except for GCNT2, which is associated with better prognosis when expressed at high levels (Figure 5c). We then examined overall survival probabilities in OC patients stratified by high versus low expression levels of the most prognostically relevant genes. Altogether, the data demonstrated that elevated expression of a subset of EMT genes correlated with reduced OS, suggesting that activation of mesenchymal pathways contributes to a more aggressive clinical course. Conversely, higher expression of select epithelial-associated genes was associated with improved OS. These data support a prognostic value for EMT transcriptional signatures in OC and highlight individual EMT molecules as potential biomarkers for patient stratification.

### 2.5. EMT Gene Dependency and In Vitro Drug Response Using OC Cell Lines

**Figure 6:**
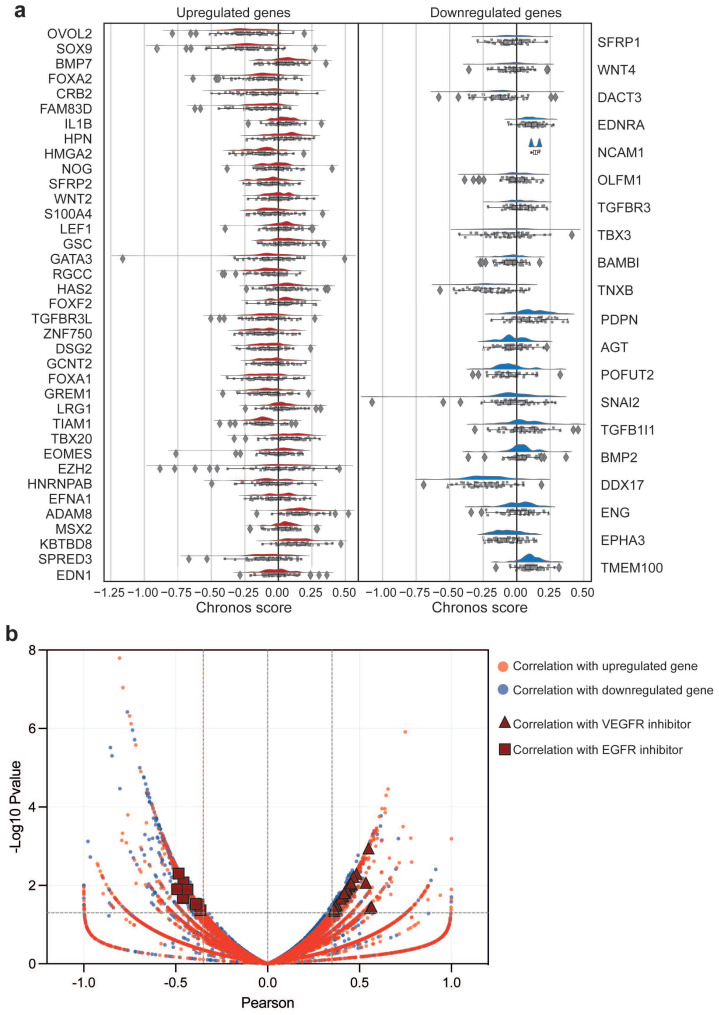
EMT Gene Dependency and In Vitro Drug Response in OC Cell Lines. (**a**) Raincloud plot depicting Chronos dependency score of EMT-associated upregulated (**left**) and downregulated (**right**) genes in OC cell lines. (**b**) Volcano plot showing the correlation between EMT gene expression and drug response in OC cell lines from the CTRpv2 database. Red dots represent correlation with upregulated genes, whilst blue dots depict correlation with downregulated genes. Triangle dots showed correlation with VEGFR inhibitors, while square dots represent correlation with EGFR inhibitors.

To determine the functional relevance of EMT-associated transcripts, we analyzed CRISPR dependency profiles in OC cell lines. Among the 1186 cell lines screened with a genome-wide CRISPR knockout library, we selected 59 OC cell lines (Supplementary Table S3). We then evaluated the effect of depleting the 37 upregulated and 20 downregulated genes associated with EMT. As shown in Figure 6a, only a few genes yield a negative Chronos score, thereby impacting OC cell survival. In particular, the genes showing the strongest dependency (Mean Chronos ≤ −0.25) are OVOL2 and SOX9 among the upregulated genes, and TNXB and DDX17 among the downregulated ones. Interestingly, none of these genes appears to have a significant impact on overall survival in OC patients. This observation suggests that the dysregulated EMT-associated genes may not play a key role in tumor cell survival processes but may instead be involved in mechanisms contributing to cancer aggressiveness, such as metastasis formation and drug response.

For this reason, taking advantage once again of the data available on the DepMap portal, we investigated whether altered expression of EMT genes could affect the response to pharmacological treatments. To this end, we analyzed the CTRPv2 portal, in which 545 drugs have been tested across more than 1700 cancer cell lines [16]. A total of 38 OC cell lines were included in the correlation analysis between the expression of the 57 dysregulated EMT genes and drug area under the curve (AUC) values (Figure 6a). Interestingly, we observed that a group of upregulated EMT-related genes showed a positive correlation (meaning that higher gene expression is associated with higher drug AUC) with a set of VEGFR inhibitors (such as Axitinib, Brivanib, AZD2171, EXEL-2880, KI8751, Lenvatinib, ABT869, MGCD-265, OSI930, AC220, RAF265, Regorafenib, SU-11248, and Tandutinib) (Figure 6b). In OC therapy, VEGF targeting represents a key therapeutic approach [17], since this factor interacts with its receptors to activate multiple signaling cascades, influencing the activation, proliferation, and migration of vascular endothelial cells, ultimately promoting tumor progression [18]. Moreover, we identified a group of genes whose expression correlates negatively (meaning that increasing gene expression is associated with lower drug AUC) with EGFR inhibitors (Afatinib, Canertinib, Erlotinib, and PD153035) (Figure 6b). EGFR inhibitors have been tested in OC patients but have shown limited effectiveness as single agents [19]. Our data suggest that this limited efficacy may be influenced by the expression levels of these specific genes.

**Figure 7:**
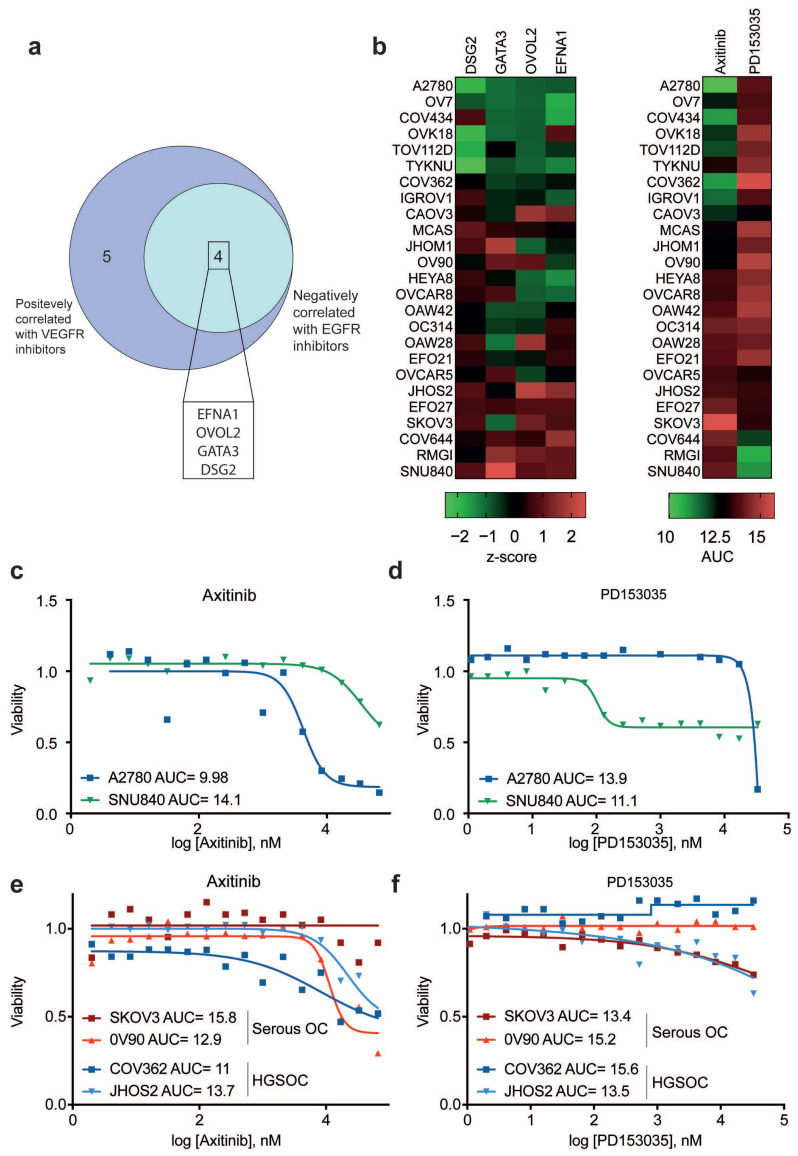
EMT-related gene signature associated with drug response in OC cell lines: (**a**) Schematic illustrating the integration of drug-response signatures to VEGFR and EGFR inhibitors and highlighting four EMT-associated genes identified as predictive markers. (**b**) Heatmaps depicting mRNA expression (z-scores) of the four ETM genes across OC cell lines together with drug response to Axitinib and PD153035 in OC cell lines (AUC values). (**c**) Dose–response curves for Axitinib in two representative OC cell lines exhibiting low (A2780) and high (SNU840) EMT-gene expression, based on AUC values obtained from CTD^2^. (**d**) Dose–response curves for PD153035 in A2780 (low-expressing) and SNU840 (high-expressing) OC cell lines, retrieved from CTD^2^. (**e**) Dose–response curves for Axitinib in two serous OC cell lines and two HGSOC cell lines exhibiting low (SKOV3 and COV362) and high (OV90 and JHOS2) EMT-related gene expression, retrieved from CTD^2^. (**f**) Dose–response curves for PD153035 in two serous OC cell lines and two HGSOC cell lines exhibiting low (SKOV3 and COV362) and high (OV90 and JHOS2) EMT-related gene expression, retrieved from CTD^2^.

Notably, when comparing genes whose expression positively correlated with the AUC of anti-VEGFR drugs to those showing negative correlation with anti-EGFR drug response, we found four genes in common, i.e., EFNA1, OVOL2, GATA3, and DSG2 (Figure 7a). We therefore analyzed the expression levels of these four genes in 25 OC cell lines, comparing them with the AUC values of a VEGFR inhibitor (Axitinib) and an EGFR inhibitor (PD153035). Interestingly, we observed that cell lines characterized by lower expression of these four genes showed low AUC for Axitinib and high AUC for PD153035, suggesting that they are much more sensitive to VEGFR inhibition than to EGFR inhibition (Figure 7b–d). Conversely, cell lines with higher expression of EFNA1, OVOL2, GATA3, and DSG2 showed high AUC for Axitinib and low AUC for PD153035 (Figure 7b–d), indicating that these cells are more sensitive to EGFR inhibition than to VEGFR inhibition. Interestingly, we observed that among the cell lines showing low expression of EFNA1, OVOL2, DSG2, and GATA3, the majority belong to the endometrioid OC subtype (A2780, OVK18, TOV112D, and IGROV1), suggesting that this subtype may be characterized by low expression of these genes and could therefore potentially benefit more from treatment with Axitinib rather than PD153035. We then investigated whether the association between the expression levels of EFNA1, OVOL2, DSG2, and GATA3, as well as the response to VEGFR and EGFR inhibitors, was also maintained in the two major OC subtypes, namely serous OC and HGSOC. As shown in Figure 7e,f, the serous OC cell line SKOV3, which exhibits high levels of the four genes, shows minimal to no response to Axitinib (AUC = 15.8) but a good response to PD153035 (AUC = 13.4), compared with another serous OC cell line with low expression of the considered genes. In contrast, another serous OC cell line with low expression of these genes, OV90, demonstrates a strong response to Axitinib (AUC = 12.9) and little to no response to PD153035 (AUC = 15.2). Similar results were observed in HGSOC cell lines. In particular, the COV362 cell line, which has low expression of EFNA1, OVOL2, DSG2, and GATA3, responds effectively to Axitinib (AUC = 11), whilst showing almost absent response to PD153035 (AUC = 15.6). Conversely, the JHOS2 HGSOC cell line, which displays high expression of the four EMT-related genes, shows a weaker response to Axitinib (AUC = 13.7) and a better response to PD153035 (AUC = 13.5) compared with COV362. Taken together, these data suggest the existence of a four-gene signature, including EFNA1, OVOL2, GATA3, and DSG2, that may be predictive of drug response and could be useful for guiding the choice of the most effective therapy.

## 3. Discussion

In this study, we provide an integrated characterization of EMT-associated genomic, transcriptomic, functional, and pharmacological alterations in OC, thereby offering a comprehensive framework to clarify how EMT contributes to tumor heterogeneity, prognosis, and therapy responsiveness. Our integrative meta-analysis of EMT-associated genes in OC provides several novel insights with potential translational relevance. By combining systematic interrogation of somatic mutations, CNVs, tumor-versus-normal transcriptomics, BRCA1/MYC stratification, CRISPR dependency screens, and drug-response correlations across TCGA and complementary datasets, we show that EMT represents a major axis of OC molecular heterogeneity, which is driven primarily by transcriptional and microenvironmental mechanisms rather than by recurrent, deterministic genomic alterations. In particular, through (1) the initial set of 160 candidate genes, (2) the identification of genes showing measurable alterations, (3) the selection of a subset of 57 genes retained after applying expression-based selection criteria and (4) the classification into 37 upregulated and 20 downregulated gene groups, we were able to identify a 4-gene upregulated signature correlating with drug response. Although we confirm that many EMT genes are altered at the genomic level (including recurrent amplifications at loci such as PTK2/FAK and prominent mutation burdens in genes such as MTOR and NOTCH4), these structural alterations rarely predict concordant changes in mRNA abundance, and this suggests that genomic amplifications or mutations alone are not sufficient to drive EMT activation in OC. Instead, our data indicate that EMT regulation relies more heavily on contextual signals such as microenvironmental cues, inflammatory and oxidative stress, and transcriptional or post-transcriptional mechanisms. This model aligns with the current concept of epithelial–mesenchymal plasticity, according to which tumor cells oscillate along a continuum of epithelial, hybrid, and mesenchymal states in response to microenvironmental stimuli rather than switching between two fixed identities [20].

Indeed, our expression analyses demonstrate a broad transcriptional deregulation of EMT genes, with 37 significantly upregulated and 20 downregulated in tumors versus normal tissues, encompassing canonical EMT transcriptional regulators, signaling intermediates, and adhesion/polarity components. When stratifying patients by BRCA1/2 mutations or MYC amplifications, we did not observe a global EMT-wide shift; however, specific EMT nodes were selectively altered, likely indicating that MYC and BRCA1/2 act as modulators of specific EMT circuits rather than as primary EMT inducers in OC. The selective, rather than global, influence of BRCA1/2 and MYC on EMT expression profiles is concordant with recent single-cell and mechanistic studies showing that homologous recombination deficiency and MYC deregulation reshape tumor microenvironmental interactions and transcriptional networks in a context-dependent fashion [21].

From a clinical standpoint, a key finding is the identification of 18 EMT-associated genes significantly correlated with overall survival, supporting the notion that only a discrete subset of EMT components—not the entire EMT program—possesses prognostic relevance. Examples include CRB2, RGCC, ADAM8, and IL1B, whose overexpression is associated with poor prognosis, and FOXA2, BMP7, and EZH2, whose upregulation is showed to be correlated with improved survival.

Furthermore, our integration of CRISPR dependency data indicates that only a minority of EMT-related genes are essential for basal OC cell fitness in vitro, and those that are important define actionable vulnerabilities whose perturbation may enhance cellular sensitivity to existing therapies. Consistent with prior evidence linking EMT and chemoresistance in OC, we also detect correlations between EMT transcriptional activity and sensitivity to clinically relevant agents—mainly inhibitors of the VEGFR and EGFR pathways, as well as patterns associated with PARP inhibitor response—supporting the concept that EMT-related condition influences therapeutic responsiveness and could inform patient stratification [22]. Indeed, our drug-response meta-analysis highlights a potential four-gene expression signature (EFNA1, OVOL2, GATA3, and DSG2) that further refines these associations. Cell lines with higher expression of this gene set exhibited increased AUC values for Axitinib but decreased AUC values for the EGFR inhibitor PD153035, indicating preferential sensitivity to EGFR blockade over VEGFR inhibition. Notably, our analysis revealed that most of the cell lines, with reduced EFNA1, OVOL2, DSG2, and GATA3 expression, are classified within the endometrioid OC subtype. This observation implies that low expression of these genes may represent a distinctive molecular feature of this subtype, potentially indicating a greater therapeutic sensitivity to Axitinib compared with PD153035.

While our study provides an integrated view of EMT-associated alterations in OC, certain points merit consideration. Most of our findings are derived from in silico analyses, in vitro CRISPR screens and transcriptomic correlations, which offer valuable predictive insights but do not yet establish causality for specific EMT-related genes in tumor progression, therapy resistance, or clinical outcomes. Moreover, although our results highlight the importance of transcriptional and microenvironmental cues in regulating EMT, these contextual signals are not fully captured in cell line models, which may limit the direct translational relevance of some observations. EMT is also a dynamic and reversible process, and static transcriptomic snapshots may not fully reflect the temporal plasticity of epithelial–mesenchymal states in tumors. Finally, despite the integration of multiple public datasets, patient heterogeneity and differences in clinical background are not to be underestimated. In addition, as the TCGA serous ovarian cystadenocarcinoma cohort predominantly consists of HGSOC, other histological subtypes are underrepresented, precluding statistically robust histotype-specific analyses.

Taken together, our findings offer a conceptual and methodological foundation for future validation in independent clinical cohorts and for functional studies aimed at determining whether incorporating EMT status into patient stratification may ultimately guide the selection of more effective, individualized therapeutic strategies in OC.

## 4. Materials and Methods

### 4.1. Ovarian Cancer Patient’s Data

OC samples were retrieved from The Cancer Genome Atlas database (https://www.cancer.gov/ccg/research/genome-sequencing/tcga, accessed on 1 November 2025), while normal bulk whole-ovary tissue samples were from the GTEX database (https://gtexportal.org/home/, accessed on 1 November 2025). TCGA sample IDs for OC biopsies and normal tissues were downloaded from the Xena database [23] (accessed in 1 November 2025), considering 398 samples with mutation and CNV data, and 419 samples with transcriptomic data. The list of considered samples is reported in Supplementary Table S2. No overlapping samples were included. Slovins’s formula was used to calculate the minimum sample size from the OC population in the TCGA dataset.

### 4.2. EMT Gene List

The EMT gene list was retrieved from the GSEA website at the following link: https://www.gsea-msigdb.org/gsea/msigdb/human/geneset/GOBP_EPITHELIAL_TO_MESENCHYMAL_TRANSITION.html (accessed on 1 November 2025). Among 190 genes, only coding genes were considered; miRNA and lncRNA were excluded from subsequent analyses. The 160 queried coding genes, listed in Supplementary Table S2, belong to the EMT collection from the GOBP_EPITHELIAL_TO_MESENCHYMAL_TRANSITION database under the accession code GO:0001837.

### 4.3. Alteration Frequency Analysis

Genomic alteration analysis for the selected OC patient cohort was conducted with the SRplot tool (https://www.bioinformatics.com.cn/srplot [24], accessed in 1 November 2025). Copy-number alterations (amplifications and deep deletions), small-scale mutations (in-frame, missense, splice-site, and truncating), and structural variants from whole-genome or whole-exome sequencing datasets were included. EMT-associated genes of interest were queried across individual samples.

### 4.4. Gene Expression Analysis

Differential expression analysis between OC and normal sample counterparts has been conducted using RNA-sequencing data from the TCGA Pan-Cancer Atlas (TCGA serous ovarian cystadenocarcinoma, which is almost exclusively composed of HGSOC samples) and the GTEX database, retrieved from UCSC Xena (https://xena.ucsc.edu/, accessed in 1 November 2025). Differential analysis has been performed using the limma voom method. Gene expression levels for EMT-related genes were normalized and expressed as Log2 (TPM + 1). Single-gene comparison was performed using GraphPad PRISM (version 10.1). Statistical analysis was performed using one-way ANOVA with post hoc testing for multiple comparisons. Comparisons with *p*-values ≤ 0.05 were considered statistically significant.

### 4.5. Overall Survival Analysis

Associations between EMT-gene expression and overall survival were evaluated using Kaplan–Meier survival analysis and Hazard Ratio calculation (HR). HR and Logrank P have been downloaded from KM plotter (https://kmplot.com/analysis/, accessed on 1 November 2025), which performs a univariate Cox regression analysis with gene expression dichotomized into high and low groups by a cut-off between the lower and upper quartiles. Overall survival data for OC patients were obtained through cBioPortal for Cancer Genomics (accessed in 1 November 2025). Survival probability curves were generated with GraphPad Prism v10.1.1.

### 4.6. Dependency Analysis

CRISPR-based genome-wide loss-of-function screening data from OC cell lines (DepMap 25Q3 + score; Chronos) (https://depmap.org/portal/, accessed in 1 November 2025) were used to evaluate gene dependency scores for EMT-associated genes. Perturbation effects are reported as Chronos dependency scores, with more negative scores reflecting stronger dependencies. Data were visualized as raincloud plots using R (version 4.4.2) custom scripts.

### 4.7. Gene Expression and Drug Sensitivity Analysis

Correlations between ETM-gene expression and drug response across OC cell lines were assessed using DepMap (public 25Q3 expression dataset and CTD^2^ AUC drug-response dataset; https://depmap.org/portal/, accessed in 1 November 2025). Pearson correlation coefficients were calculated between EMT gene expression and area-under-the-curve (AUC) values for approved or investigational compounds. Correlation values with |r| > 0.35 and *p* ≤ 0.05 were retained for downstream interpretation. Significant correlations were visualized in Volcano plots using GraphPad Prism v10.1.1.

### 4.8. Statistical Analysis

GraphPad Prism 10.1.1 was used to calculate *p*-values by one-way ANOVA for multiple comparisons. A *p*-value < 0.05 was considered statistically significant and is indicated in the Figures as follows: * *p* < 0.05, ** *p* < 0.01, *** *p* < 0.001, **** *p* < 0.0001. Groups showing no statistical significance are unmarked.

## Data Availability

Data supporting the findings of this study are openly available and were downloaded from the cBioportal database (https://www.cbioportal.org) (accessed on 1 November 2025), the Xena database (https://xena.ucsc.edu/) (accessed in 1 November 2025), and the DepMap database (https://depmap.org/portal/) (accessed in 1 November 2025).

## Supplementary Materials

The following supporting information can be downloaded at: https://www.mdpi.com/article/10.3390/ijms27052149/s1.

## Abbreviations

The following abbreviations are used in this manuscript:

OC	Ovarian Cancer
HGSOC	High-grade serous ovarian cancer
EMT	Epithelial–mesenchymal transition
TCGA	The Cancer Genome Atlas
GTEx	Genotype-tissue expression
CNV	Copy number variations
FC	Fold change
HR	Hazard ratio
AUC	Area under the curve
